# Genetic Characteristics and Preservative Tolerance of Spoilage Microorganisms in Daily Chemical Products

**DOI:** 10.3390/microorganisms14071528

**Published:** 2026-07-13

**Authors:** Xia Wen, Shuyao Zhang, Di Huang, Aiting Su, Yiwen Chen, Hongbing Tao, Yankun Xu, Guifang Zhang, Xiaobao Xie

**Affiliations:** 1 Guangdong Provincial Key Laboratory of Microbial Culture Collection and Application, State Key Laboratory of Applied Microbiology Southern China, Guangdong Detection Center of Microbiology, Institute of Microbiology, Guangdong Academy of Sciences, Guangzhou 510070, China; wenxia@gdim.cn (X.W.);; 2 Guangdong Demay Biological Technology Co., Ltd., Guangzhou 510663, China

**Keywords:** daily chemical products, *Burkholderia cepacia* complex, *Pseudomonas aeruginosa*, multilocus sequence typing, preservative tolerance

## Abstract

Daily chemical products (DCPs), including cosmetics and cleaning products, are widely used in everyday life but are vulnerable to microbial contamination during production, storage, and use. Such contamination can cause product spoilage, reduce product quality, and pose potential health risks to consumers. In this study, we performed a comprehensive surveillance of spoilage microorganisms isolated from 185 abnormal DCPs batches collected from 2019 to 2021, followed by phenotypic identification, multilocus sequence typing (MLST), phylogenetic analysis, and preservative susceptibility testing. A total of 1655 microbial isolates were recovered from raw materials, the production environment, and finished products. The most prevalent contaminants in finished products were *Burkholderia* spp. and *Pseudomonas* spp., and correlation analysis suggested that raw materials were a major reservoir of contamination. Species-level identification showed that the *Burkholderia cepacia* complex (Bcc) and *Pseudomonas aeruginosa* were the dominant spoilage bacteria. MLST analysis of 41 Bcc isolates identified 15 sequence types (STs), including six novel STs, while 23 *P. aeruginosa* isolates were assigned to nine STs. Among these, Bcc ST621 and *P. aeruginosa* ST2230 were the most frequently detected lineages. Preservative challenge assays showed that several Bcc and *P. aeruginosa* isolates exhibited tolerance to Kathon at or above the maximum permitted concentration. These findings indicate that raw materials are an important source of microbial contamination in DCPs and that Bcc and *P. aeruginosa* lineages with elevated preservative tolerance may contribute to product spoilage. Our results support the inclusion of Bcc in routine microbial monitoring of DCPs and highlight the need for improved raw material control and preservative efficacy evaluation.

## 1. Introduction

Daily chemical products (DCPs) are formulated consumer products used in daily personal care, household cleaning, hygiene, fragrance, and related applications, including cosmetics, shampoos, body washes, hand soaps, detergents, disinfectant wipes, and surface-cleaning products. Because DCPs differ widely in formulation, intended use, regulatory classification, and brand-specific safety or efficacy claims, the term is used here as a broad category. Many DCPs contain water, surfactants, plant-derived extracts, oils, proteins, polysaccharides, and other organic compounds that may support microbial survival or growth when preservation systems or hygienic controls are insufficient. Microbial contamination may occur during raw material storage, production, filling, transportation, retail display, or consumer use [1]. Microbial contamination can lead to product spoilage, discoloration, odor formation, viscosity changes, reduced shelf life, and, in some cases, consumer health risks [2]. Recent studies have reported microbial contamination in cosmetic products. A study of 50 cosmetic products collected from shops in the Mecca region of Saudi Arabia showed that most products were contaminated with bacteria or fungi, with low-quality brands exhibiting higher and more diverse contamination than high-quality brands. Commonly detected microorganisms included *Staphylococcus aureus*, *Escherichia coli*, *Streptococcus pneumoniae*, *Staphylococcus epidermidis*, *Bacillus subtilis*, *Pseudomonas aeruginosa*, and fungi such as *Aspergillus*, *Penicillium*, and *Rhizopus* species [3]. In addition, a study of 60 unsealed cosmetic testers collected from shopping malls in Saudi Arabia detected *S. epidermidis*, *Candida albicans*, *Cutibacterium acnes*, *P. aeruginosa*, and *S. aureus*, suggesting that repeatedly used cosmetic products may act as reservoirs or vehicles for microorganisms, including opportunistic pathogens [4]. Therefore, microbial contamination of cosmetics and related DCPs remains an important quality and safety issue.

To reduce microbial contamination, many countries and regions have established regulatory requirements and microbiological limits for cosmetics and related products, including the European Union Cosmetics Regulation, U.S. FDA guidance, and the Chinese Technical Specifications for the Safety of Cosmetics [5,6,7,8]. Despite these measures, microbial contamination remains frequently reported in commercial products. Previous studies have shown that a substantial proportion of cosmetics and personal care products exceed acceptable microbial limits, and some contaminated products have been associated with opportunistic infections, especially in individuals with compromised skin barriers or reduced immunity [9,10,11,12]. These findings underscore the need for more effective microbial surveillance and control strategies in DCPs production.

Among the microorganisms associated with DCPs contamination, members of the *Burkholderia cepacia* complex (Bcc) and *Pseudomonas aeruginosa* are of particular concern. Many hospital infections originated from the use of cosmetics contaminated by *Burkholderia cepacia*, such as reports of an outbreak of *Burkholderia cepacia* infection among hospitalized patients caused by contaminated colored cosmetic contact lenses, mouthwash or no-rinse cleansing foam product [13,14,15], and an outbreak of *Burkholderia cepacia* in the intensive care unit caused by contaminated moisturizing lotion [16]. Infection caused by *Burkholderia cepacia* is related to the use of contaminated liquid soap or damp towels in hospitals [17,18]. *P. aeruginosa* was found to be present in cosmetics such as mascara, lip balm, facial cleanser and body or facial cream. These microorganisms are classified as opportunistic pathogens, capable of causing skin irritation and infections, especially when the skin is damaged [12]. Recent studies have shown that the cosmetic contact lenses are contaminated with *Pseudomonas aeruginosa*, which poses a risk of causing microbial keratitis [19]. Zhang et al. reported a rare case of green nail syndrome caused by a co-infection with *P. aeruginosa* and the Bcc in a woman with a history of manicures and frequent domestic exposure to water and detergents [20].

Although several studies have reported microbial contamination in DCPs [12], most have focused on descriptive surveys of contamination rates or on individual isolates. Large-scale studies integrating source analysis, molecular typing, and preservative susceptibility testing remain limited, particularly for Bcc and *P. aeruginosa* from daily chemical manufacturing systems. In addition, the genetic diversity and lineage distribution of these organisms in DCPs have not been sufficiently characterized, and the relationship between clonal types and preservative tolerance remains unclear.

To address these gaps, we conducted a comprehensive investigation of spoilage microorganisms isolated from abnormal DCPs samples showing abnormal appearance or suspected quality deterioration collected from manufacturing enterprises in Guangdong Province over a three-year period. In this study, such samples were selected because they showed visible or sensory deviations suggestive of possible spoilage, but they were not considered microbiologically contaminated until viable microorganisms were detected and identified by culture-based and molecular methods. We aimed to (i) characterize the distribution of contaminating microorganisms in raw materials, production environments, and finished products; (ii) identify the major spoilage bacteria at the species level; (iii) analyze the genetic diversity of Bcc and *P. aeruginosa* by MLST and phylogenetic analysis; and (iv) evaluate the susceptibility of major clonal types to commonly used preservatives. Because microbial contamination may be influenced by product type, formulation, raw material quality, manufacturing environment, manufacturer-specific quality control practices, and regional conditions, this study was intended to provide insight into microbial risks associated with abnormal DCPs samples rather than to estimate the general contamination prevalence of all DCPs or brands. Our findings provide new insight into the microbial ecology of DCP contamination and may help improve contamination prevention and preservative management in the industry.

## 2. Materials and Methods

### 2.1. Isolation and Identification of Spoilage Microorganisms in Daily Chemical Products

From 2019 to 2021, a total of 185 batches of daily chemical product DCPs-related samples showing abnormal appearance, suspected quality deterioration, or potential microbiological risk were collected from manufacturing enterprises in Guangdong Province, China. These samples were derived from three source categories: raw materials, production environments, and finished products. Raw material samples referred to formulation ingredients or intermediate materials used in DCPs manufacturing, which may carry microorganisms through contaminated water, plant- or animal-derived components, surfactants, additives, or other nutrient-containing substances. Production environment samples referred to samples collected from manufacturing-related environments, such as workshops, equipment surfaces, product-contact areas, water systems, pipelines, storage areas, or other sites associated with production, where microorganisms may persist and contribute to cross-contamination. According to the sampling method of Korzekwa et al. [21], environments samples were collected using a sterile swab and were suspended in 1 mL of sterile saline (Macklin Biochemical Co., Ltd, Shanghai, China), followed by serial dilutions. Finished product samples referred to final DCPs showing abnormal appearance or suspected quality deterioration, including facial masks, shampoos, body wash products, essence liquids, hand sanitizers, and laundry detergents. Abnormal appearance or suspected quality deterioration was defined as visible or sensory deviations from expected product characteristics, including turbidity, precipitation, phase separation, discoloration, abnormal odor, gas formation, viscosity changes, package swelling or leakage, or other signs suggestive of possible spoilage. Hand sanitizers and laundry detergents were included because products with antimicrobial, inhibitory, or cleaning functions are not necessarily sterile and may still be contaminated during manufacturing, storage, transportation, or consumer use. Sample details (source, sampling time, brand, batch number, manufacturing, and expiry date) were recorded, and the samples were transported to the laboratory under sterile conditions, stored at 4 °C, and processed within 24 h.

Microbiological examination was performed according to the Safety and Technical Standards for Cosmetics (2015 edition) issued by the National Medical Products Administration of China [6]. Briefly, one gram or one milliliter from each sample was aseptically transferred to 10 mL sterile saline (0.85% NaCl) and homogenized for 10 min. Then, 1 mL of the diluted sample was transferred aseptically into a sterile Petri dish, and 1 mL was transferred into 9 mL sterile saline to prepare a 1:100 dilution. This serial dilution procedure was repeated up to 10^−6^. After dilution, melted lecithin-Tween 80 nutrient agar (Huankai Microbial Sci.&Tech. Co., Ltd., Guangzhou, China) and potato dextrose agar (PDA) (Huankai Microbial Sci.&Tech. Co., Ltd., Guangzhou, China), both cooled to 45 °C, were poured into the dishes. Sterile saline was used as a blank control. Plates were incubated at 37 °C for 48 h for bacteria and at 28 °C for 7 days for fungi, after which colony counts were recorded.

Distinct colonies were selected according to colony morphology, including colony size, shape, color, margin, elevation, opacity, and surface texture. Preliminary phenotypic characterization was performed based on morphological characteristics, Gram staining, microscopic observation, and physiological and biochemical profiles obtained using the API identification system, with reference to the Common Bacteria System Identification Manual and the manufacturer’s instructions for the API system (BioMérieux, Shanghai, China). The purified isolates were then subjected to molecular identification based on 16S rRNA gene sequencing for bacteria and ITS sequencing for fungi.

Total DNA was extracted using a rapid nucleic acid extraction kit (TaKaRa, Tokyo, Japan) according to the manufacturer’s instructions. For bacterial isolates, the 16S rRNA gene was amplified using the universal primers 27F (5′-AGAGTTTGATCATGGCTCAG-3′) and 1492R (5′-TAGGGTTACCTTGTTACGACTT-3′). For fungal isolates, the ITS region was amplified using ITS1 (5′-TCCGTAGGTGAACCTGCGG-3′) and ITS4 (5′-TCCTCCGCTTATTGATATGC-3′). PCR conditions were as follows: initial denaturation at 95 °C for 5 min; 35 cycles of denaturation at 94 °C for 50 s, annealing at 54 °C for bacteria or 55 °C for fungi for 40 s, and extension at 72 °C for 1 min 30 s; followed by a final extension at 72 °C for 10 min. PCR products were sequenced by Huada Gene Technology Co., Ltd. (Shenzhen, China). Sequence data were analyzed and compared with reference sequences in the NCBI BLAST database (National Center for Biotechnology Information, Bethesda, MD, USA).

### 2.2. MLST Test of the Burkholderia cepacia Complex

Multilocus sequence typing (MLST) was performed for *Burkholderia cepacia* complex (Bcc) isolates. Seven housekeeping genes were selected, including *atpD* (ATP synthase β-chain), *gltB* (glutamate synthase large subunit), *gyrB* (DNA gyrase subunit B), *recA* (recombinase A), *lepA* (GTP-binding protein), *phaC* (acetyl-CoA dehydrogenase), and *trpB* (tryptophan synthase subunit B). Primer sequences were obtained from the Bcc PubMLST database (https://pubmlst.org/organisms/burkholderia-cepacia-complex) (accessed on 28 June 2025) and are listed in Table S1. PCR amplification was performed in a 50 µL reaction mixture containing 25 µL of 2× Rapid Taq Master Mix, 2.0 µL of template DNA (10 ng/µL), 2.0 µL of forward primer (10 pmol/µL), 2.0 µL of reverse primer (10 pmol/µL), and 19 µL of ddH_2_O. The cycling conditions were as follows: initial denaturation at 96 °C for 1 min; 30 cycles of denaturation at 96 °C for 1 min, annealing at 58 °C for 1 min, and extension at 72 °C for 2 min; followed by a final extension at 72 °C for 5 min. PCR products were purified using a DNA purification kit and sequenced by Beijing Henglu Huada Genomics Technology Co., Ltd. (Guangzhou Branch, China). Forward and reverse sequences of each locus were aligned, trimmed, and edited using SeqMan II software (Lasergene package). Allele numbers and sequence types (STs) were assigned using the Bcc MLST database.

### 2.3. MLST Test of Pseudomonas aeruginosa

MLST analysis of *Pseudomonas aeruginosa* was conducted using seven housekeeping genes: *acsA* (acetyl coenzyme A synthetase), *aroE* (shikimate dehydrogenase), *guaA* (GMP synthase), *mutL* (DNA mismatch repair protein), *nuoD* (NADH dehydrogenase I chain C/D), *ppsA* (phosphoenolpyruvate synthase), and *trpE* (anthranilate synthetase component I). Primer sequences were obtained from the *P. aeruginosa* PubMLST database (https://pubmlst.org/organisms/pseudomonas-aeruginosa/) (accessed on 28 June 2025) and are listed in Table S2. PCR was performed in a 50 µL reaction mixture containing 25 µL of 2× Rapid Taq Master Mix, 2.0 µL of template DNA (10 ng/µL), 2.0 µL of forward primer (10 pmol/µL), 2.0 µL of reverse primer (10 pmol/µL), and 19 µL of ddH_2_O. The thermal cycling conditions were as follows: initial denaturation at 96 °C for 1 min; 30 cycles of denaturation at 96 °C for 1 min, annealing at 55 °C for 1 min, and extension at 72 °C for 1 min; followed by a final extension at 72 °C for 5 min. PCR products were purified and sequenced by Beijing Huada Genomics Technology Co., Ltd. (Guangzhou Branch, China). Sequence assembly and editing were performed using SeqMan II software. Allele numbers and STs were determined using the *P. aeruginosa* PubMLST database.

### 2.4. Preservative Susceptibility Testing of Major Spoilage Bacteria

The preservative tolerance assay was performed based on the method described by Rushton et al. [22]. Preservatives, Kathon (MIT/CMIT, methylisothiazolinone/methylchloroisothiazolinone, 3:1) and DMDMH (dimethylol dimethyl hydantoin), were evaluated against the major spoilage bacteria isolated from daily chemical products. A Kathon stock solution (0.14%) was prepared by dilution with sterile distilled water. A DMDMH stock solution (1.2%) was prepared by dissolving 1.2 g of DMDMH in 10 mL of sterile distilled water, followed by filtration through a 0.22 μm sterile membrane filter. The filtered DMDMH solution was stored at 4 °C until use. Working solutions were prepared by serial dilution to obtain final concentrations of 0.0025%, 0.00125%, 0.000625%, 0.0003125%, 0.000156%, and 0.0000781% for Kathon, and 1.2%, 0.6%, 0.4%, 0.2%, 0.1%, and 0.05% for DMDMH. Bcc and *Pseudomonas aeruginosa* isolates were cultured to the logarithmic growth phase and adjusted to 10^6^ CFU/mL in LB broth. For each assay, 50 μL of preservative solution was added to each well of a microplate, followed by 50 μL of bacterial suspension. Each concentration was tested in triplicate. Wells containing sterile water instead of bacterial suspension were used as negative controls, and wells containing bacterial suspension without preservative were used as positive controls. The microplate was incubated at 37 °C for 24 h, then 10 μL of culture from wells showing no visible growth was inoculated onto nutrient agar plates and incubated at 37 °C overnight.

Preservative susceptibility testing was performed in triplicate independent biological replicates. The minimum inhibitory concentration (MIC) was defined as the lowest preservative concentration that completely inhibited visible microbial growth after incubation, and the minimum bactericidal concentration (MBC) was defined as the lowest concentration resulting in no colony recovery after subculture onto preservative-free medium. When replicate results differed by one dilution, the higher concentration was used for conservative reporting.

### 2.5. Statistical Analysis

All data were analyzed using GraphPad Prism version 9.0 (GraphPad Software, San Diego, CA, USA). Pearson correlation analysis was performed using SPSS version 19.0 (IBM Corp., Armonk, NY, USA). Statistical significance was defined as *p* < 0.05. Multiple sequence alignment and phylogenetic tree construction were both performed using MEGA 12. The phylogenetic analysis was conducted using the neighbor-joining algorithm based on genetic distance.

## 3. Results

### 3.1. The Distribution of Spoilage Microorganisms in DCPs

A total of 1655 spoilage microorganism isolates were recovered from 185 batches of abnormal daily chemical product samples collected between 2019 and 2021. These isolates originated from raw materials (*n* = 376), the production environment (*n* = 538), and finished products (*n* = 741). At the genus level, the dominant microorganisms in raw materials were *Ralstonia* spp. (71 isolates, 18.9%), *Burkholderia* spp. (63, 16.7%), *Bacillus* spp. (54, 14.3%), *Enterobacter* spp. (31, 8.1%), *Pseudomonas* spp. (26, 6.9%), and *Halomonas* spp. (10, 2.6%).

In the production environment, the predominant genera were *Staphylococcus* spp. (79 isolates, 14.6%), *Bacillus* spp. (64, 11.8%), *Micrococcus* spp. (61, 11.3%), *Moraxella* spp. (37, 6.8%), *Pseudomonas* spp. (31, 5.7%), *Ralstonia* spp. (25, 4.6%), *Kocuria* spp. (23, 4.2%), *Burkholderia* spp. (22, 4.0%), *Acinetobacter* spp. (16, 2.9%), *Penicillium* spp. (12, 2.2%), *Exiguobacterium* spp. (10, 1.8%), and *Klebsiella* spp. (10, 1.8%).

**Figure 1:**
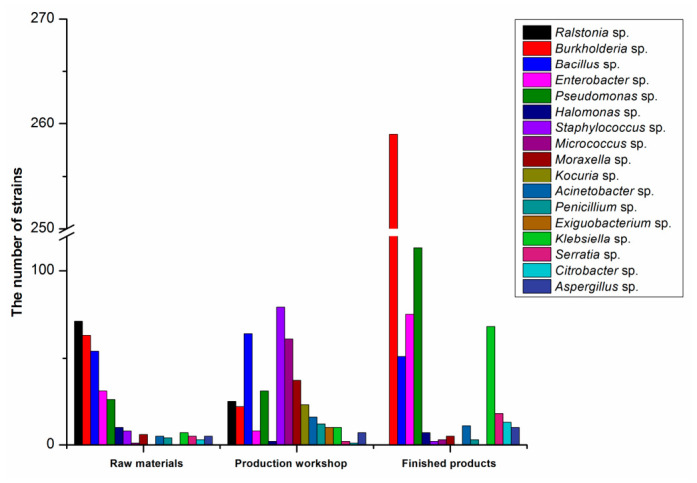
The distribution of microorganisms in raw materials, production workshops and finished products.

In finished products, *Burkholderia* spp. (259 isolates, 34.9%) were the most frequently recovered, followed by *Pseudomonas* spp. (113, 15.2%), *Enterobacter* spp. (75, 10.0%), *Klebsiella* spp. (68, 9.1%), *Bacillus* spp. (51, 6.8%), *Serratia* spp. (18, 2.4%), *Citrobacter* spp. (13, 1.7%), *Acinetobacter* spp. (11, 1.4%), and *Aspergillus* spp. (10, 1.3%) (Figure 1).

**Figure 2:**
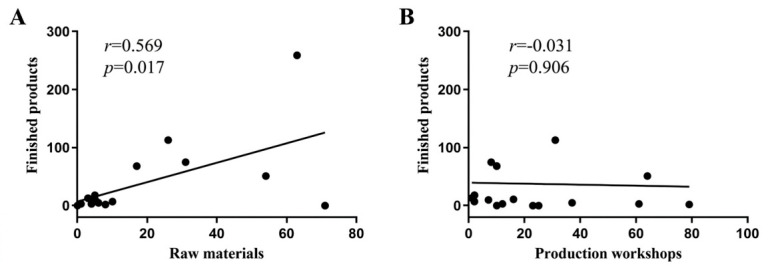
Correlation analysis of microorganisms in finished products, raw materials and production workshops. (**A**) finished products and raw materials. (**B**) finished products and production workshops.

Pearson correlation analysis of the 17 most abundant microbial taxa detected in raw materials (Figure 2), the production environment, and finished products revealed a significant positive correlation between microorganisms in finished products and raw materials (*p* < 0.05, r = 0.569). In contrast, the correlation between microorganisms in finished products and the production environment was not significant (*p* > 0.05, r = 0.031). The significant correlation between microbial contamination in raw materials and finished products suggests a possible association between these sample types. However, correlation analysis alone cannot determine contamination pathways or prove that raw materials were the direct source of contamination in finished products.

**Figure 3:**
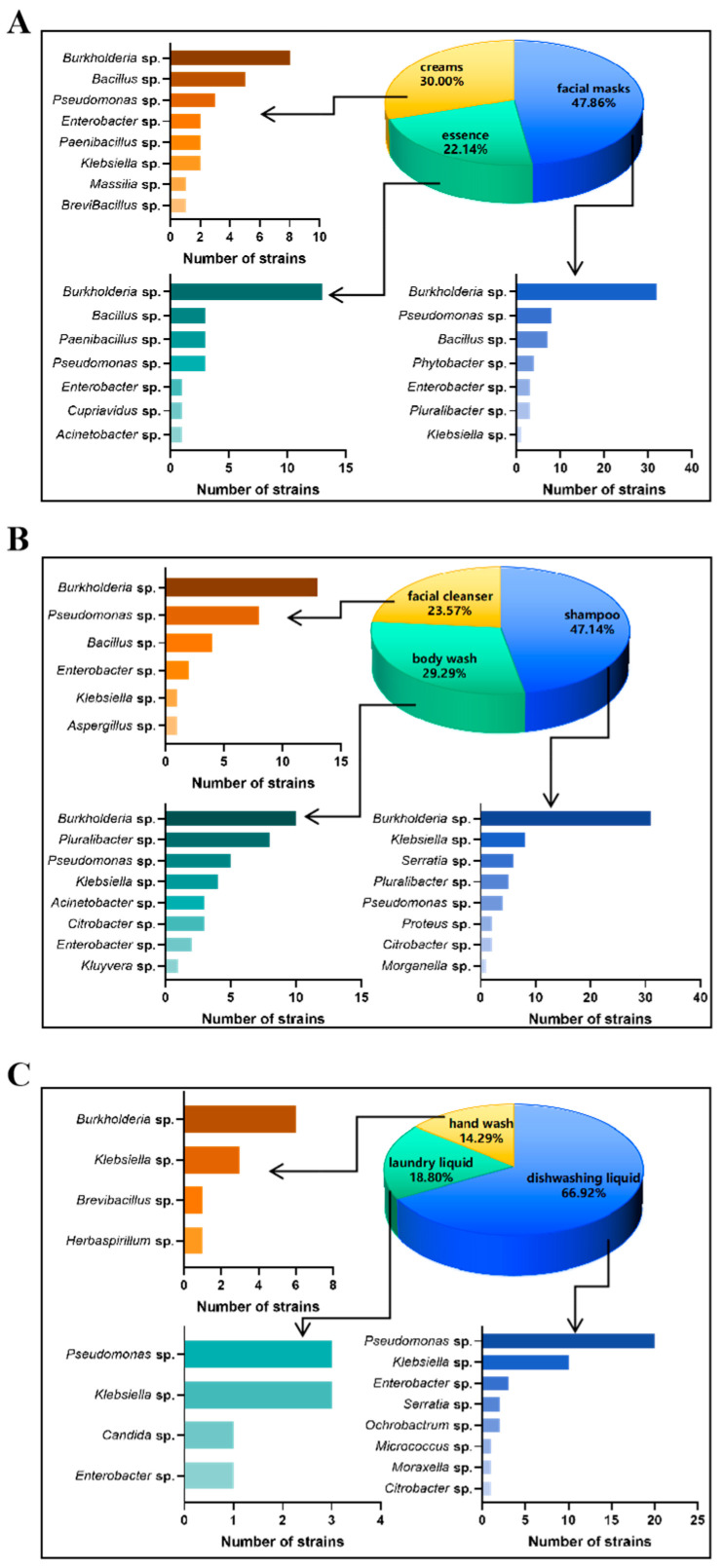
Distribution of microbe genus in three types of DCPs. (**A**) Skin care products. (**B**) Body cleaning products. (**C**) Cleaning products.

When product types were analyzed separately, spoilage microorganisms in skin care products (facial masks, creams, and essences), body cleansing products (shampoo, body wash, and facial cleanser), and washing products (dishwashing liquid, laundry detergent, and hand wash) showed distinct distribution patterns (Figure 3). In skin care products, *Burkholderia* spp., *Bacillus* spp., and *Pseudomonas* spp. were predominant, with facial masks showing the highest contamination frequency (47.86%). In body cleansing products, *Burkholderia* spp., *Pluralibacter* spp., and *Klebsiella* spp. were the dominant genera, and shampoo showed the highest contamination frequency (47.14%). In washing products, *Burkholderia* spp. and *Pseudomonas* spp. were most common, followed by *Klebsiella* spp. Dishwashing liquid exhibited the highest contamination frequency (66.92%). Overall, *Burkholderia* spp., *Pseudomonas* spp., *Klebsiella* spp., and *Bacillus* spp. were the most common spoilage microorganisms in daily chemical products.

**Figure 4:**
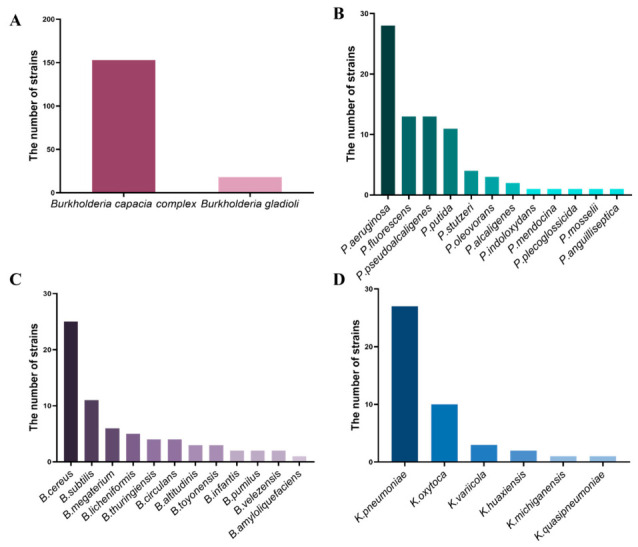
Distribution of microbial species at the genus level in DCPs. (**A**) *Burkholderia* spp. (**B**) *Pseudomonas* spp. (**C**) *Bacillus* spp. (**D**) *Klebsiella* spp.

Species-level identification of these major genera further showed that the *Burkholderia cepacia* complex (Bcc) accounted for 153 of 171 *Burkholderia* isolates (89.47%). Among *Pseudomonas* isolates, *P. aeruginosa* was the most common species (28 isolates, 35.44%), followed by *P. fluorescens* and *P. pseudoalcaligenes* (13 isolates each, 16.46%), and *P. putida* (11 isolates, 13.92%). Among *Bacillus* isolates, *B. cereus* was the dominant species (25 isolates, 36.76%), followed by *B. subtilis* (11 isolates, 16.18%) and *B. megaterium* (6 isolates, 8.82%). Among *Klebsiella* isolates, *K. pneumoniae* was predominant (27 isolates, 61.36%), followed by *K. oxytoca* (10 isolates, 22.73%) (Figure 4).

### 3.2. MLST Analysis of Major Spoilage Microorganisms

#### 3.2.1. MLST Analysis of the *Burkholderia cepacia* Complex

A total of 41 Bcc isolates representing different manufacturers and product types were selected for MLST. These isolates were derived from skin care products (facial masks, moisturizing lotions, toners, essences, and wipes) and body cleansing products (shampoo, body wash, and hand sanitizer), originating from 18 manufacturers. Sequencing of the seven housekeeping genes generated 15 STs (Tables S3 and S4).

**Figure 5:**
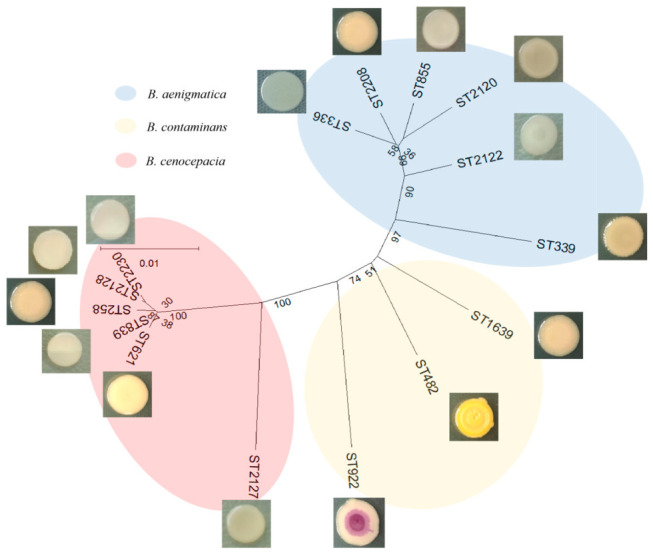
Phylogenetic tree and phenotypic characteristics of MLST gene series based on Bcc.

Several allele profiles could not be matched to existing MLST database entries and were therefore submitted as novel alleles, resulting in the assignment of six new STs(ST2208, ST2120, ST2122, ST2127, ST2128, ST2230) (Tables S3 and S4). Phylogenetic analysis based on concatenated sequences of the seven housekeeping genes grouped the Bcc isolates into three species: *B. aenigmatica*, *B. contaminans*, and *B. cenocepacia* (Figure 5). Among these, *B. aenigmatica* and *B. contaminans* appeared to be more closely related to each other than to *B. cenocepacia*.

**Figure 6:**
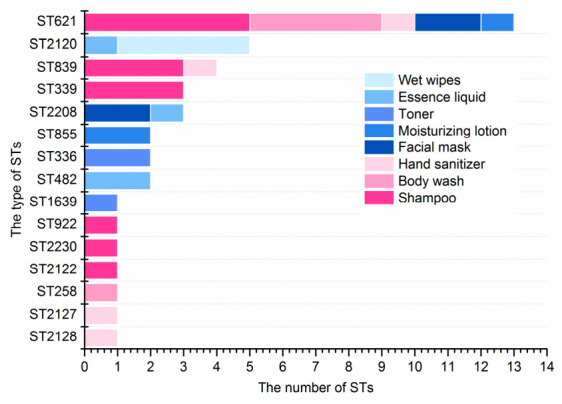
Distribution of different ST type strains in Bcc in DCPs.

The distribution of Bcc species and STs differed by product category (Figure 6). In washing products, *B. cenocepacia* was the most common species (58.54%), and ST621 was the most abundant ST (31.7%). In skin care products, *B. aenigmatica* predominated (57.14%), and ST2120 was the most frequent ST (30.77%). Distinct colony morphologies were also observed among different STs on TSA plates. Yellow colonies were predominant overall, ST482 formed bright yellow colonies, whereas ST922 produced purple colonies with spreading growth from the center toward the edge, suggesting ST-associated phenotypic variation.

#### 3.2.2. MLST Analysis of *Pseudomonas aeruginosa*

**Figure 7:**
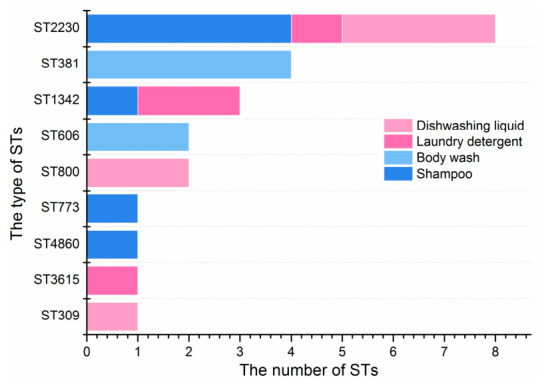
Distribution of different ST types of *P. aeruginosa* in samples.

MLST analysis was performed on 23 *P. aeruginosa* isolates recovered from shampoo, body wash, laundry detergent, and dishwashing liquid samples (Table S5). Nine STs were identified: ST2230, ST381, ST1342, ST606, ST800, ST773, ST4860, ST3615, and ST309 (Figure 7). *P. aeruginosa* ST2230 was the most prevalent type, accounting for 34.78% (8/23) of isolates, and was recovered from shampoo, laundry detergent, and dishwashing liquid samples. ST381 was the second most common ST and was isolated exclusively from body wash samples.

**Figure 8:**
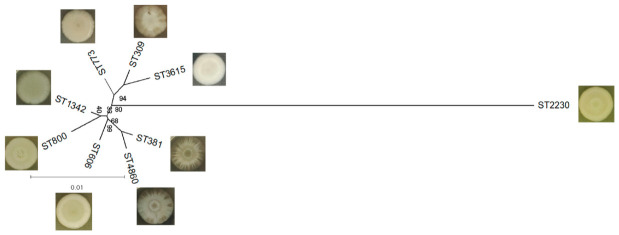
Phylogenetic tree based on MLST steward gene tandem sequence of *P. aeruginosa*.

Phylogenetic analysis based on concatenated MLST loci suggested that ST2230 was the predominant sequence type among the *P. aeruginosa* isolates and appeared centrally positioned in the MLST-based neighbor-joining tree (Figure 8), indicating that these isolates may have diversified from a common genetic background.

### 3.3. Inhibitory Effect of Preservatives on Bcc

**Figure 9:**
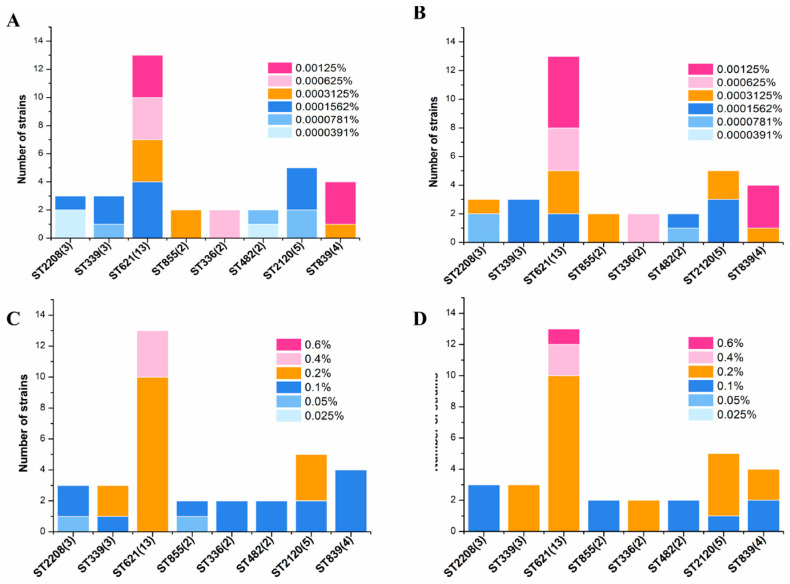
Inhibitory effects of Kathon and DMDMH on Bcc. (**A**) MIC of Kathon on Bcc. (**B**) MBC of Kathon on Bcc. (**C**) MIC of DMDMH on Bcc. (**D**) MBC of DMDMH on Bcc.

Eight Bcc STs that were detected more than twice were selected for preservative susceptibility testing: ST2208, ST339, ST621, ST855, ST336, ST482, ST2120, and ST839 (Figure 9). According to the Cosmetic Safety Technical Specifications, the maximum permitted concentration of Kathon (MIT/CMIT) is 0.0015%. Our results showed that the MIC values of Kathon for ST621 (9 strains), ST855 (2 strains), ST336 (2 strains), and ST839 (4 strains) were above 0.0015%. In addition, the MBC values of Kathon for ST2280 (1 strain), ST621 (11 strains), ST855 (2 strains), ST336 (2 strains), ST2120 (2 strains), and ST839 (4 strains) were also above 0.0015%, indicating strong tolerance of most Bcc isolates to Kathon.

DMDMH is another commonly used preservative in daily chemical products, with a maximum permitted concentration of 0.6%. In the present study, 0.4% DMDMH inhibited all tested Bcc isolates, while 0.6% DMDMH completely eliminated them. These results indicate that DMDMH was more effective against Bcc than Kathon under the experimental conditions used.

### 3.4. Inhibitory Effect of Preservatives on P. aeruginosa

**Figure 10:**
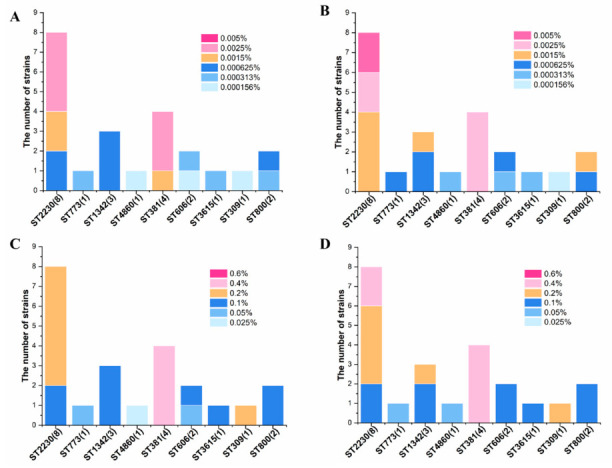
Inhibitory effects of Kathon and DMDMH on *P. aeruginosa*. (**A**) MIC of Kathon on *P. aeruginosa*. (**B**) MBC of Kathon on *P. aeruginosa*. (**C**) MIC of DMDMH on *P. aeruginosa*. (**D**) MBC of DMDMH on *P. aeruginosa*.

A total of 23 *P. aeruginosa* isolates representing 11 STs were evaluated for preservative susceptibility (Figure 10). The MIC of Kathon was 0.0015% for ST2230 (2 isolates) and ST381 (1 isolate), and 0.0025% for ST2230 (4 isolates) and ST381 (3 isolates). Regarding the MBC, 0.0015% was observed for ST2230 (4 isolates), ST1342 (1 isolate), and ST880 (1 isolate); 0.0025% for ST2230 (2 isolates), ST1342 (1 isolate), and ST381 (4 isolates); and 0.005% for ST2230 (2 isolates). These results indicate that the tolerance of different *P. aeruginosa* STs to Kathon varied substantially, with ST2230 and ST381 showing the strongest tolerance.

For DMDMH, the MIC was 0.2% for ST2230 (6 isolates) and ST800 (1 isolate), while the MIC was 0.4% for ST381 (4 isolates). The MBC of DMDMH was 0.4% for ST2230 (2 isolates) and ST381 (4 isolates). Although ST2230 and ST381 exhibited relatively high tolerance to DMDMH, all tested *P. aeruginosa* isolates were inhibited or killed at concentrations below the maximum permitted concentration of 0.6%. Therefore, DMDMH was broadly effective against the *P. aeruginosa* isolates examined in this study.

## 4. Discussion

The present study focused on abnormal DCPs samples showing visible or sensory signs of suspected quality deterioration rather than randomly selected products from the retail market. Therefore, the microorganisms recovered in this study should be interpreted as spoilage-associated organisms from high-risk samples, not as an estimate of the overall microbial contamination prevalence of all DCPs. Microbial contamination of DCPs can occur during raw material handling, production, storage, transportation, and consumer use, and may be influenced by product type, formulation characteristics, production water, equipment sanitation, personnel hygiene, packaging, and storage conditions. Potential sources of microorganisms in DCPs manufacturing include raw materials of plant, animal, or synthetic origin, production water, processing equipment, production personnel, and the surrounding manufacturing environment. In the present study, microorganisms detected in raw materials were positively correlated with those in finished products, whereas no significant correlation was found between finished products and the production environment. These findings suggest that raw materials may be an important reservoir of contamination in the sampled enterprises. Singh et al. detected fungal (*Aspergillus*) contamination in plant raw materials, and some of them may produce highly toxic substances, such as aflatoxins [23]. Tassaneeyakul et al. investigated the distribution of aflatoxins in plant raw materials using different analytical methods and found that additives such as starch may be the cause of fungal contamination in plant raw materials leading to toxin production [24]. Consequently, microbial quality control of raw materials should be regarded as a critical step in preventing product spoilage and ensuring product safety. Although microbial recovery from raw materials was correlated with that from finished products, this finding should be interpreted cautiously. Pearson correlation analysis indicates statistical association but does not establish causality or contamination routes. Definitive source attribution would require paired batch-level sampling, longitudinal tracking, source-tracking approaches, or genomic comparisons.

Among the spoilage microorganisms recovered in this study, Bcc and *P. aeruginosa* were the most prevalent Gram-negative bacteria. Both taxa are well known for their environmental adaptability, biofilm-forming capacity, and reduced susceptibility to preservatives and antimicrobials [25,26]. Bcc, in particular, is an important industrial contaminant and a frequent cause of product recalls because it can survive in low-nutrient environments and develop adaptive tolerance under preservative pressure [22,27]. The frequent detection of *Pseudomonas* spp., especially *P. aeruginosa*, in abnormal DCPs samples is consistent with previous reports of contamination in cosmetics, cosmetic ingredients, and manufacturing environments. *P. aeruginosa* is also highly versatile, with strong colonization capacity and intrinsic resistance mechanisms that allow it to persist on abiotic surfaces and in aqueous products [28,29]. Amir et al. isolated an alkali-tolerant *P. aeruginosa* strain from cosmetic foundation and confirmed its identity by biochemical tests and 16S rRNA gene sequencing, indicating that this species can survive under stress conditions in cosmetic matrices [30]. Zabielska et al. isolated *P. aeruginosa* from body balm and showed that these isolates had hydrophobic, aggregative, and adhesive properties that may facilitate colonization of abiotic surfaces [31]. In addition, Korzekwa et al. isolated *P. aeruginosa* strains from cosmetic manufacturing environments, including production lines, sodium laureth sulfate pipelines, calendula extract, and shower gel, and demonstrated their ability to form biofilms on materials commonly used in cosmetic production equipment [21]. The frequent detection of Bcc and *P. aeruginosa* in DCPs may be associated with several previously reported microbial traits, such as environmental adaptability, tolerance to nutrient-limited conditions, and biofilm-forming ability. However, these mechanisms were not specifically assessed in the present study. Therefore, further mechanistic investigations are required to obtain more definitive conclusions regarding their contribution to the occurrence and persistence of these microorganisms in DCPs.

MLST is a rapidly developing method in molecular biology with high resolution capabilities. The principle of this method is to amplify internal fragments of multiple housekeeping genes and determine their sequences using Sanger sequencing [32]. This method has been used for epidemiological monitoring and evolutionary research of various bacteria [33]. MLST analysis revealed considerable genetic diversity among the Bcc and *P. aeruginosa* isolates. Among Bcc isolates, *B. aenigmatica*, *B. contaminans*, and *B. cenocepacia* were the major species, and several novel STs were identified, which are the most common Bcc species in industrial products and the environment [34,35]. The predominance of ST621 in washing products and ST2120 in skin care products indicates that certain clonal lineages may be particularly adapted to specific product categories. At the same time, ST621 was found to be the most prevalent ST type, which is consistent with our previous report [36]. Other researchers also found ST621 strains among 42 BCC isolates obtained from tertiary hospitals [37]. The isolation of this pathogenic ST type from cosmetic products indicates that this pathogen requires a rich gene pool and multiple pathogenic strategies to develop the capacity to circumvent the physical barriers and innate defense mechanisms of the host [38]. The phylogenetic relationship observed among the Bcc species suggests close relatedness between *B. aenigmatica* and *B. contaminans*, with *B. cenocepacia* forming a distinct lineage. This pattern is consistent with the complex evolutionary background of Bcc and highlights the need for strain-level surveillance in industrial settings. For *P. aeruginosa*, ST2230 was the most common ST and was recovered from multiple washing and cleaning products. ST2230 was the predominant sequence type among the *P. aeruginosa* isolates. In the MLST-based neighbor-joining tree, isolates belonging to ST2230 clustered with several other sequence types, suggesting genetic relatedness based on the analyzed housekeeping genes. This finding is notable because ST2230 has been reported from both environmental and clinical sources [39], suggesting that it may possess broad ecological adaptability. The repeated detection of ST2230 in daily chemical products indicates that certain *P. aeruginosa* lineages may be particularly suited to survival in manufacturing and product environments.

Preservative susceptibility testing further demonstrated that several Bcc isolates tolerated Kathon concentrations at or above the maximum permitted level. This is highly relevant from an industrial perspective because Kathon is one of the most widely used preservatives in rinse-off products. The ability of Bcc to tolerate Kathon may contribute to its frequent recovery from spoiled products and may help explain preservative failures in certain formulations. Among Bcc species, *B. lata* strains exhibited tolerance to MIT (methylisothiazolinone), which may be associated with the activity of efflux pumps. Upregulation or sustained expression of multiple efflux systems could facilitate active export of the preservative, thereby reducing its intracellular accumulation and increasing tolerance [22]. Meanwhile, previous studies have suggested that tolerance of *B. cepacia* to MIT/CMIT may be related to alterations in outer membrane proteins and decreased cell envelope permeability, which restrict preservative uptake. In addition, enhanced peroxide-sensing responses and increased expression of antioxidant enzymes may contribute to the ability of these bacteria to withstand isothiazolinone-induced oxidative stress [40]. Collectively, these mechanisms may underlie preservative tolerance in Bcc isolates. However, the mechanism of Kathon tolerance in Bcc remains unclear and warrants further in-depth investigation. Some isolates belonging to ST2230 and ST621 showed relatively elevated MIC/MBC values. The preservative susceptibility analysis was limited by the uneven number of isolates across sequence types and, for some STs, small sample sizes. Therefore, the observed differences in MIC/MBC values among sequence types should be regarded as descriptive trends rather than definitive lineage-associated differences. Future studies using larger and more balanced isolate collections, together with standardized replicate testing and statistical comparisons, are needed to determine whether preservative tolerance is significantly associated with specific genetic lineages.

In contrast, DMDMH showed better activity against Bcc, completely inhibiting growth at 0.4% and killing all tested isolates at 0.6%. Nevertheless, the actual efficacy of DMDMH in commercial formulations may be affected by formulation complexity, pH, surfactants, packaging, and storage conditions. Therefore, preservative performance should be evaluated in the context of the full product matrix rather than solely by in vitro testing. From a quality-control perspective, routine microbial monitoring should not only focus on conventional indicator organisms but should also include Bcc, particularly in products that contain aqueous phases or are prone to preservative failure. More broadly, these results support the need for source tracking, strain-level surveillance, and preservative challenge testing tailored to the microbial ecology of daily chemical manufacturing.

Among *P. aeruginosa* isolates, ST2230 and ST381 exhibited the strongest tolerance to Kathon, and even the maximum permitted concentration did not completely eradicate all isolates. This observation is consistent with previous reports that *P. aeruginosa* can withstand isothiazolone-based preservatives [41]. DMDMH was more effective, and all tested *P. aeruginosa* isolates were inhibited or killed at concentrations below the legal upper limit. These results indicate that preservative efficacy varies considerably by strain and by chemical class, underscoring the importance of incorporating representative spoilage organisms into challenge tests.

Several limitations should be considered when interpreting the findings of this study. First, the samples were collected from manufacturing enterprises in Guangdong Province and consisted mainly of abnormal or suspected deteriorated DCPs-related samples, rather than randomly selected commercial products. Therefore, the results should not be used to estimate the overall contamination prevalence of all DCPs, brands, or regions. Second, microbial contamination in DCPs may be influenced by multiple factors, including product formulation, water content, pH, preservative system, packaging, raw material control, production water quality, equipment cleaning, and environmental sanitation. These factors were not systematically evaluated in the present study. Third, although isolates were recovered from raw materials, production environments, and finished products, paired batch-level sampling and high-resolution source-tracking methods such as whole-genome sequencing were not performed. Thus, the exact contamination sources and transmission routes could not be definitively determined. Future studies combining systematic sampling, formulation and process information, environmental monitoring, and genome-based source tracking are needed to better clarify contamination pathways and improve microbial control strategies in DCPs manufacturing.

## 5. Conclusions

In conclusion, this study provides a comprehensive characterization of spoilage microorganisms recovered from abnormal DCPs-related samples collected from manufacturing enterprises in Guangdong Province. The frequent recovery of microorganisms from raw material-related samples suggests that raw materials may represent an important potential contributor to microbial contamination in DCPs production, although definitive source attribution requires systematic paired sampling and longitudinal tracking. Bcc and *P. aeruginosa* were identified as the predominant spoilage microorganisms, indicating that these opportunistic pathogens deserve greater attention in routine microbial surveillance of DCPs.

MLST analysis revealed substantial genetic diversity among the isolates. *B. cenocepacia* ST621 and *B. aenigmatica* ST2120 were among the most common Bcc lineages, while ST2230 was the predominant sequence type among *P. aeruginosa*. The identification of several novel STs suggests that DCPs-associated contaminants include genetically diverse and previously uncharacterized lineages. Preservative susceptibility testing further showed that some Bcc and *P. aeruginosa* isolates had strong tolerance to Kathon, whereas DMDMH was more effective under the tested conditions.

These findings highlight the importance of strengthening raw-material surveillance, environmental monitoring, and preservative efficacy evaluation in DCPs production. Routine microbial testing programs should consider including Bcc, in addition to currently monitored organisms, to improve contamination prevention, quality control, and consumer safety.

## Data Availability

The original contributions presented in this study are included in the article/Supplementary Materials. Further inquiries can be directed to the corresponding author.

## Supplementary Materials

The following supporting information can be downloaded at: https://www.mdpi.com/article/10.3390/microorganisms14071528/s1, Table S1: Primers used for MLST typing of Bcc; Table S2: Primers used for MLST typing of *Pseudomonas aeruginosa*; Table S3: MLST typing of Bcc in skin care products; Table S4: MLST typing of Bcc in body cleaning products; Table S5: MLST typing of *Pseudomonas aeruginosa* in washing and cleaning products.
