# Supplementary material for: Genetic Characteristics and Preservative Tolerance of Spoilage Microorganisms in Daily Chemical Products
Source: Microorganisms. 2026 Jul 13;14(7):1528. doi: 10.3390/microorganisms14071528 (PMC13414242; doi:10.3390/microorganisms14071528)
Supplement: Supplementary file 1 [file microorganisms-14-01528-s001.zip › microorganisms-4372173-supplementary.pdf]

Table S1. Primers used for MLST typing of Bcc

Locus	Forward primer（5’-3’）	Reverse primer（5’-3’）	Amplification length
atpD	GTTCATCTGGCCGTACAC	AACTGACGCTCGAAGTCC	443bp
gltB	CTTCTTCTTCGTCGCCGA	TTGCCGACGTAGTCGTTG	400bp
gyrB	ATCGTGATGACCGAGCTG	CGTTGTAGCTGTCGTTCC	454bp
recA	TGACCGCCGAGAAGAGCAA	GACCGAGTCGATGACGAT	393bp
lepA	GGCATCAAGGAACTGACG	CTGCGGCATGTACAGGTT	397bp
phaC	AGACGGCTTCAAGGTGGT	ACACGGTGTTGACCGTCA	385bp
trpB	CTGGGTCACGAACATGGA	CCGAATGCGTCTCGATGA	301bp

Table S2. Primers used for MLST typing of *Pseudomonas aeruginosa*

Locus	Forward primer（5'-3'）	Reverse primer（5'-3'）	Amplification length
acsA	ACCTGGTGTACGCCTCGCTGAC	GACATAGATGCCCTGCCCCTTGAT	390bp
aroE	TGGGGCTATGACTGGAAACC	TAACCCGGTTTTGTGATTCCTACA	498bp
guaA	CGGCCTCGACGTGTGGATGA	GAACGCCTGGCTGGTCTTGTGGTA	373bp
mutL	CCAGATCGCCGCCGGTGAGGTG	CAGGGTGCCATAGAGGAAGTC	442bp
nuoD	ACCGCCACCCGTACTG	TCTCGCCCATCTTGACCA	366bp
ppsA	GGTCGCTCGGTCAAGGTAGTGG	GGGTTCTCTTCTTCCGGCTCGTAG	370bp
mutL	GCGGCCCAGGGTCGTGAG	CCCGGCGCTTGTTGATGGTT	443bp

Table S3. MLST typing of Bcc in skin care products

Strain number	manufacturers	Source of the strain	*atpD*	*gltB*	*gyrB*	*recA*	*lepA*	*phaC*	*trpB*	ST	Strain name
BC-01	A1	facial masks	726	967	1421	146	214	162	882	new(2208)	*B. aenigmatica*
BC-02	A1	facial masks	726	967	1421	146	214	162	882	new(2208)	*B. aenigmatica*
BC-03	B1	facial masks	184	224	301	200	219	53	210	339	*B. aenigmatica*
BC-04	B1	facial masks	184	224	301	200	219	53	210	339	*B. aenigmatica*
BC-05	B1	facial masks	184	224	301	200	219	53	210	339	*B. cenocepacia*
BC-06	C1	facial masks	15	11	481	14	11	6	147	621	*B. cenocepacia*
BC-07	C1	facial masks	15	11	481	14	11	6	147	621	*B. cenocepacia*
BC-08	D1	moisturizing lotions	180	403	600	146	415	317	217	855	*B. cenocepacia*
BC-09	D1	moisturizing lotions	180	403	600	146	415	317	217	855	*B. cenocepacia*
BC-10	E1	moisturizing lotions	15	11	481	14	11	6	147	621	*B. cenocepacia*
BC-11	F1	toners	538	572	864	489	668	406	529	1639	*B. contaminans*
BC-12	G1	toners	182	215	297	146	214	162	218	336	*B. aenigmatica*
BC-13	G1	toners	182	215	297	146	214	162	218	336	*B. aenigmatica*
BC-14	H1	essences	726	967	1421	146	214	162	882	new(2208)	*B. aenigmatica*
BC-15	H1	essences	180	201	1351	778	887	310	851	new(2120)	*B. aenigmatica*
BC-16	I1	essences	151	192	245	152	1158	173	151	482	*B. contaminans*
BC-17	I1	essences	151	192	245	152	158	173	151	482	*B. contaminans*
BC-18	J1	wipes	180	201	1351	778	887	310	851	new(2120)	*B. aenigmatica*
BC-19	J1	wipes	180	201	1351	778	887	310	851	new(2120)	*B. aenigmatica*
BC-20	K1	wipes	180	201	1351	778	887	310	851	new(2120)	*B. aenigmatica*
BC-21	K1	wipes	180	201	1351	778	887	310	851	new(2120)	*B. aenigmatica*

Table S4. MLST typing of Bcc in body cleaning products

Strain number	manufacturers	Source of the strain	*atpD*	*gltB*	*gyrB*	*recA*	*lepA*	*phaC*	*trpB*	STs	Strain name
BCC-22	A2	shampoo	15	11	481	14	11	6	147	621	*B. cenocepacia*
BCC-23	A2	shampoo	15	11	481	14	11	6	147	621	*B. cenocepacia*
BCC-24	A2	shampoo	361	325	640	382	431	329	330	922	*B. contaminans*
BCC-25	B2	shampoo	15	64	506	14	11	6	147	new(2230)	*B. cenocepacia*
BCC-26	B2	shampoo	15	11	481	14	11	6	147	621	*B. cenocepacia*
BCC-27	B2	shampoo	180	390	579	354	214	310	210	new(2122)	*B. aenigmatica*
BCC-28	C2	shampoo	15	11	487	14	11	6	79	839	*B. cenocepacia*
BCC-29	C2	shampoo	15	11	487	14	11	6	79	839	*B. cenocepacia*
BCC-30	C2	shampoo	15	11	487	14	11	6	79	839	*B. cenocepacia*
BCC-31	D2	shampoo	15	11	481	14	11	6	147	621	*B. cenocepacia*
BCC-32	D2	shampoo	15	11	481	14	11	6	147	621	*B. cenocepacia*
BCC-33	D2	body wash	15	11	481	14	11	6	147	621	*B. cenocepacia*
BCC-34	D2	body wash	15	11	481	14	11	6	147	621	*B. cenocepacia*
BCC-35	E2	body wash	15	11	481	14	11	6	147	621	*B. cenocepacia*
BCC-36	E2	body wash	15	11	481	14	11	6	147	621	*B. cenocepacia*
BCC-37	E2	body wash	15	192	236	14	11	6	79	258	*B. cenocepacia*
BCC-38	F2	hand sanitizer	107	155	343	67	11	41	252	new(2127)	*B. cenocepacia*
BCC-39	F2	hand sanitizer	131	11	1353	14	11	6	79	new(2128)	*B. cenocepacia*
BCC-40	G2	hand sanitizer	15	11	481	14	11	6	147	621	*B. cenocepacia*
BCC-41	G2	hand sanitizer	15	11	487	14	11	6	79	839	*B. cenocepacia*

Table S5. MLST typing of *Pseudomonas aeruginosa* in washing and cleaning products

Strain number	manufacturers	Source of the strain	*acsA*	*aroE*	*guaA*	*mutL*	*nuoD*	*ppsA*	*trpE*	STs
PA-01	A3	shampoo	87	198	114	123	86	93	146	2230
PA-02	A3	shampoo	87	198	114	123	86	93	146	2230
PA-03	A3	shampoo	87	198	114	123	86	93	146	2230
PA-04	A3	shampoo	87	198	114	123	86	93	146	2230
PA-05	B3	shampoo	5	4	5	5	5	7	8	773
PA-06	C3	shampoo	1	5	26	3	1	10	3	1342
PA-07	D3	shampoo	11	5	83	11	50	3	7	4860
PA-08	E3	body wash	11	20	1	65	4	4	10	381
PA-09	E3	body wash	11	20	1	65	4	4	10	381
PA-10	E3	body wash	11	20	1	65	4	4	10	381
PA -11	E3	body wash	11	20	1	65	4	4	10	381
PA-12	F3	body wash	23	5	57	30	1	4	3	606
PA-13	F3	body wash	23	5	57	30	1	4	3	606
PA-14	G3	laundry detergent	13	13	17	3	1	6	7	3615
PA-15	H3	laundry detergent	87	198	114	123	86	93	146	2230
PA-16	I3	laundry detergent	1	5	26	3	1	10	3	1342
PA-17	I3	laundry detergent	1	5	26	3	1	10	3	1342
PA-18	J3	dishwashing liquid	13	8	9	3	1	17	15	309
PA-19	K3	dishwashing liquid	17	22	11	3	3	15	3	800
PA-20	K3	dishwashing liquid	17	22	11	3	3	15	3	800
PA-21	L3	dishwashing liquid	87	198	114	123	86	93	146	2230
PA-22	L3	dishwashing liquid	87	198	114	123	86	93	146	2230
PA-23	L3	dishwashing liquid	87	198	114	123	86	93	146	2230
